# A bibliometric study of the nasopharyngeal cancer immunotherapy knowledge map

**DOI:** 10.1097/MD.0000000000037763

**Published:** 2024-04-19

**Authors:** Huanhuan Xie, Wenjing Liu, Mi Yang

**Affiliations:** a Department of Oncology, Nanjing Drum Tower Hospital Clinical College of Nanjing University of Chinese Medicine, Nanjing, Jiangsu, China.

**Keywords:** bibliometric studies, CiteSpace, immunotherapy, nasopharyngeal carcinoma, VOSviewer

## Abstract

Nasopharyngeal carcinoma (NPC) is one of the most common malignant tumors, and stages III and IV are frequently diagnosed. In recent years, immunotherapy has achieved remarkable results in recurrent/metastatic NPC, and many studies related to immunotherapy for NPC have been published. However, to date, no relevant bibliometric studies have been published. The trends and research focus on NPC immunotherapy are analyzed in this study through bibliometric analysis, which is conducive to better understanding the status quo and future trends of immunotherapy for NPC. The Web of Science Core Collection was used to collect literature on NPC immunotherapy. These publications were analyzed using bibliometric methods from the aspects of country/region, institution, author (co-cited author), journal (co-cited journal), references, and keywords to determine the research focus and trends in the field. A total of 510 English studies were published between January 1, 2000 and September 1, 2023. The number of articles published increased rapidly in 2016. China ranked first in the number of publications (n = 254), followed by the United States (n = 127). Sun Yat-sen University had the largest number of publications (n = 74). In terms of authors, Comoli P is the most cited author among the co-cited authors. The journal publishing the largest number of studies on NPC immunotherapy is *Frontiers in Oncology* (impact factor (2022) = 4.7). Five of the top 10 highly cited publications came from China. Keyword analysis reveals that infiltrating lymphocytes, PD-L1, and the tumor microenvironment are recent research focuses on nasopharyngeal cancer immunotherapy. Immunotherapy research for nasopharyngeal cancer is a recent trend. Nasopharyngeal cancer immunotherapy research has mainly focused on immune checkpoint inhibitors and the tumor microenvironment. Notably, China has made significant contributions to this field.

## 1. Introduction

Nasopharyngeal carcinoma (NPC) is a malignant tumor that occurs in the head and neck. It has different etiologies, histopathology, and epidemiology,^[1]^ NPC is not included in the discussion of head and neck cancers. In 2020, there were 133,354 new NPC cases and 80,008 deaths were reported worldwide.^[2]^ At the time of initial diagnosis, more than 75% of NPC patients are diagnosed with stage III or IV.^[3]^ Recurrence and/or metastasis (R/M) leads to treatment failure in about 20% to 30% of patients with advanced NPC.^[4]^ Currently, there is no satisfactory treatment for R/M NPC. The median overall survival (OS) of these patients was 20 to 30 months.^[5,6]^ Even in high-income countries, the 5-year survival rate of patients with stage IV disease is <40%.^[7,8]^ Immunocheckpoint inhibitors (ICI) possess the capacity to impede the binding of immunosuppressive signals to their corresponding ligands and ultimately enhance the antitumor immune effect of the body. Fortunately, many immune cells have been identified in NPC.^[9]^ At present, ICI have become a promising treatment for R/M NPC, and many related clinical trials are underway. To understand the current research hotspots and potential research sites in this field, we conducted a scientometric analysis of immunotherapy for nasopharyngeal cancer.

Bibliometric analysis employs citation counts as an assessment metric to evaluate research quality, and can analyze trends in research outputs through statistical and qualitative assessment.^[10]^ It provides a way to understand trends in each field and rank academic groups and individuals by objectively assessing their contributions through a comprehensive analysis of citations to countries and other aspects.^[11–13]^ To date, bibliometric studies on NPC immunotherapy knowledge maps have not been published. The aim of this study was to evaluate existing research and potential future areas in the field of NPC immunotherapy between 2000 and 2023.

## 2. Materials and methods

### 2.1. Data collection

**Figure 1.**
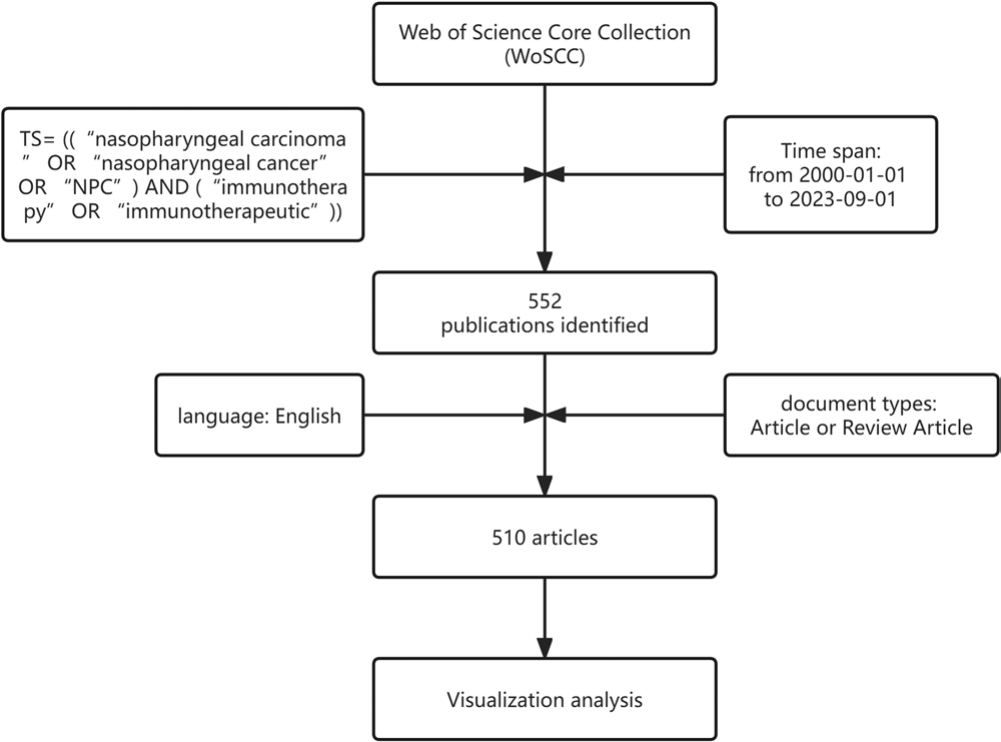
The flow chart of research steps.

On September 23, 2023, using the Web of Science Core Collection (WoSCC), we explored the literature pertaining to NPC immunotherapy from January 1, 2000 to September 1, 2023. The Science Citation Index-Expanded and Social Science Citation Index were used as the data sources, restricting the article type to “article” or “review.” We used TS = ((“nasopharyngeal carcinoma” OR “nasopharyngeal cancer” OR “NPC”) AND (“immunotherapy” OR “immunotherapeutic”)) for the search. According to the above requirements, the 2 authors independently carry out the above operations and download the required data in TXT format, including title, author, country, magazine, keywords, and reference-related information. All data downloads were completed on September 23, 2023 to avoid potential bias due to frequent database updates. Differing viewpoints were resolved through discussions and third parties. A flowchart of the research steps is presented in Figure 1.

### 2.2. Analyze

Using R (version 4.3.2), we analyzed the frequency of publication by year and top 10 countries in terms of publishing volume and generated descriptive statistics. Currently, CiteSpace and VOSviewer are frequently used in bibliometric analyses. Several researchers have used this strategy to assess their respective research areas.^[14,15]^ We choose to use these 2 software for data analysis. The data collected from WoSCC were then imported into VOSviewer software (version 1.6.19), a free computer program commonly employed for bibliometric analysis and the creation of visually intuitive maps. VOSviewer’s distinctive features enable the representation of large-scale bibliometric data in a comprehensible manner.^[16]^ The overlay network maps about co-authorship of different countries, the network maps about co-citation analysis of authors and the network, overlay, and density map about co-occurrence analysis of keywords were obtained using VOSviewer. Keyword citation bursts were analyzed using CiteSpace (version6.2. R4) citation bursts because it allows for the analysis of citation bursts, enabling the display of burst intensity and duration.^[13]^

## 3. Results

### 3.1. Data collection

**Figure 2.**
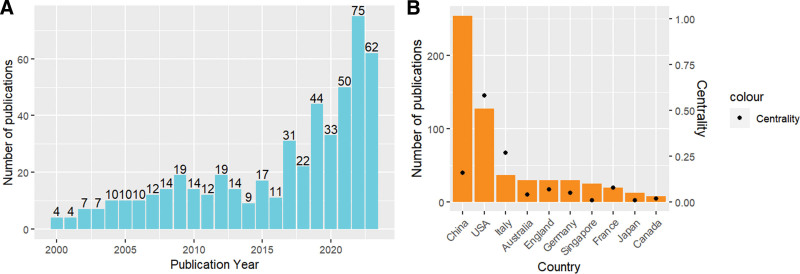
(A) From 2000 to 2023, trends in the number of publications in NPC immunotherapy research. (B) Top 10 countries with published documents. The column height represents the number of publications, and the black dot represents the centrality.

By searching the WoSCC database, 510 studies on NPC immunotherapy, published between January 1, 2000 and September 1, 2023, were obtained. Figure 2A shows a significantly increasing trend in the number of studies on NPC immunotherapy since 2016. By analyzing the data until 2022, the average annual growth rate of articles published between 2016 and 2022 was calculated to be 54.88%. Significantly, the annual growth rates in 2017, 2019, 2021, and 2022 have exceeded or equaled 50%.

### 3.2. Analysis of countries and organizations

**Figure 3.**
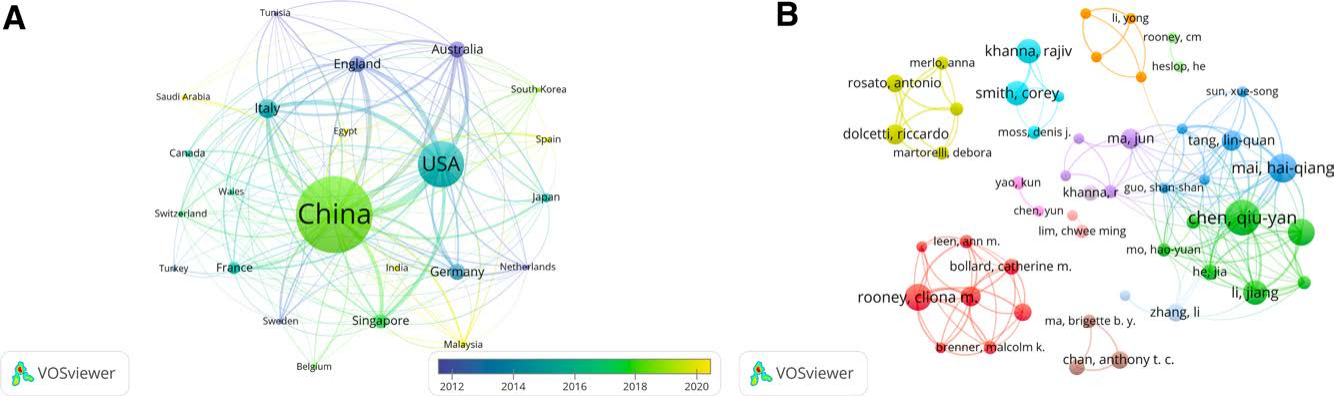
(A) Overlay map of countries/regions in NPC immunotherapy research. The size of points is in direct proportion to the number of published documents, and the lines between points represent the cooperative links between countries. Purple: early time, yellow: near time. (B) The network map of authors. Different colors represent different clusters, lines between points represent cooperation and contact, and the lack of communication between authors is obvious.

A total of 510 papers came from 51 countries/regions. Figure 2B provides a comprehensive list of the top 10 nations responsible for the largest volume of published work. China and the United States accounted for 74.71% (381/510) of all publications, with China contributing 254, and the United States contributing 127. China occupies the top spot in terms of article quantity; however, it ranks third in terms of the centrality index, boasting a specific value of 0.16. At the heart of the network, the United States (0.58) serves as an intermediary in cooperation between countries and holds substantial sway over the publishing industry, with Italy closely behind (0.27). Figure 3A visually represents the collaborative network among the 23 countries/regions that have contributed to more than 2 papers.

In Table 1, it can be observed that Sun Yat-sen University (n = 74) secured the top position among the 782 organizations in terms of article publications, while Baylor College of Medicine (n = 32) and Central South University (n = 19) followed closely behind. The high average citation count of 116 indicates that the research conducted by Texas Children’s Hospital has generated significant interest despite having published a relatively small number of articles (17).

**Table 1:** Top 10 institutions publishing research articles on immunotherapy for NPC.

Rank	Organization	Publications	Citations	Average number of citations
1	Sun Yat-sen University	74	2517	34
2	Baylor College of Medicine	32	3231	101
3	Central South University	19	678	36
4	The Chinese University of Hong Kong	17	866	51
5	Nanjing Medical University	17	434	26
6	Texas Children’s Hospital	17	1965	116
7	Queensland Institute of Medical Research	16	611	38
8	University of Birmingham	14	987	71
9	The University of Hong Kong	14	1539	110
10	National University of Singapore	13	185	14
11	Sichuan University	13	420	32

From the data in Table 1, although Texas Children’s Hospital has only 17 articles, the average number of citations per article is as high as 116, which shows that their research has received considerable attention.

### 3.3. Analysis of journals and co-cited journals

A total of NPC 250 journals has published relevant articles regarding immunotherapy for NPC. Among them, *Frontiers in Oncology* (n = 22, impact factor 2022 = 4.7) ranked first, followed by *Frontiers in Immunology* (n = 19, impact factor 2022 = 7.3). Table 2 lists the top 9 journals, of which 44.5% (4/9) were from Switzerland, followed by the United States (22.2%, 2/9), England (22.2%, 2/9), and the Netherlands (11.1%, 1/9). Among the 2686 journals involved in the references, the *Journal of Clinical Oncology* received the highest number of citations (1651 citations), followed by *Blood* (1325 citations) and *Cancer Research* (968 citations). Notably, 77.8% (7/9) of the co-cited journals were from the United States, reflecting the importance of the United States in the publication of related research on NPC immunotherapy.

**Table 2:** Top 9 journals and co-cited journal in NPC immunotherapy research.

Rank	Journal	Publications	Country	Import factor (2022)	Rank	Cited journal	Citations	Country	Import factor (2022)
1	*Frontiers in Oncology*	22	Switzerland	4.7	1	*Journal of Clinical Oncology*	1651	USA	45.2
2	*Frontiers in Immunology*	19	Switzerland	7.3	2	*Blood*	1325	USA	20.3
3	*Cancers*	12	Switzerland	5.2	3	*Cancer Research*	968	USA	11.2
4	*Cancer Immunology Immunotherapy*	11	USA	5.8	4	*Clinical Cancer Research*	789	USA	11.5
5	*Oral oncology*	11	England	4.8	5	*The New England Journal of Medicine*	747	USA	158.5
6	*Oncoimmunology*	10	USA	7.2	6	*Journal of Immunology*	724	USA	4.4
7	*Journal for Immunotherapy of Cancer*	8	England	10.9	7	*Journal of Virology*	702	USA	5.4
8	*Annals of Oncology*	7	Netherlands	50.5	8	*International Journal of Cancer*	689	Switzerland	6.4
9	*International Journal of Cancer*	7	Switzerland	6.4	9	*Annals of Oncology*	601	Netherlands	50.5

### 3.4. Analysis of authors and co-cited authors

There were 3147 authors who conducted research related to immunotherapy for NPC, of which the top 4 most published papers were Chen Qiuyan (n = 16), Mai Haiaiang (n = 13), Rooney Clionam (n = 12), and Zeng Yixin (n = 12). As shown in Table 3, among the co-cited authors, Comoli P was the most cited (185 citations), followed by Khanna R (181 citations), and Bollard CM (170 citations). The utilization of VOSviewer (Fig. 3B) enabled the exploration of author co-authorship and citation networks. Each author is represented by a point on the graph, the size of which indicates the number of documents published by the author. Additionally, the lines linking these points illustrate the co-occurrence relationships between authors. Figure 3B illustrates a significant co-occurrence pattern, indicating that highly productive authors tend to have a higher tendency to co-occur with other authors.

**Table 3:** Top 11 authors and top 10 coauthors involved in NPC immunotherapy research.

Rank	Author	Documents	Citations	Rank	Coauthor	Citations
1	Chen Qiuyan	16	844	1	Comoli P	185
2	Mai Haiqiang	13	558	2	Khanna R	181
3	Rooney Clionam	12	1540	3	Bollard CM	170
4	Zeng Yixin	12	536	4	Chan ATC	157
5	Khanna Rajiv	11	312	5	Lee AWM	151
6	Li Jiang	11	551	6	Ma BBY	148
7	Smith Corey	11	281	7	Chen YP	138
8	Dolcetti Riccardo	9	251	8	Zhang L	124
9	Heslop Helene	9	1477	9	Lee SP	122
10	Ma Jun	9	286	10	Smith C	121
11	Tang Linquan	9	385			

### 3.5. Analysis of co-cited literatures

Table 4 lists the 10 most cited articles in the literature related to NPC immunotherapy. The most co-cited article on immunotherapy research for NPC was “Treatment of nasopharyngeal carcinoma with Epstein-Barr virus-specific T lymphocytes,”^[17]^ published in *Blood* and cited 104 times. Articles with ≥90 total citations (n = 3) were published mainly in *Blood* and *Journal of Clinical Oncology*.

**Table 4:** The details of the top 10 cited references.

Rank	Citations	Title	Corresponding author	Journal	Year	IF (2022)
1	104	Treatment of nasopharyngeal carcinoma with Epstein-Barr virus-specific T lymphocytes	Heslop HE	*Blood*	2005	20.3
2	91	Cell therapy of stage IV nasopharyngeal carcinoma with autologous Epstein-Barr virus-targeted cytotoxic T lymphocytes	Comoli P	*Journal of Clinical Oncology*	2005	45.3
3	90	Antitumor activity of nivolumab in recurrent and metastatic nasopharyngeal Carcinoma: an international, multicenter study of the Mayo Clinic Phase 2 Consortium (NCI-9742)	Ma Brigette BY	*Journal of Clinical Oncology*	2018	45.3
4	89	Safety and antitumor activity of Pembrolizumab in patients with programmed death-ligand 1-positive nasopharyngeal carcinoma: results of the KEYNOTE-028 study	Hsu Chiun	*Journal of Clinical Oncology*	2017	45.3
5	70	Camrelizumab (SHR-1210) alone or in combination with gemcitabine plus cisplatin for nasopharyngeal carcinoma: results from two single-arm, phase 1 trials	Zhang Li	*Lancet Oncology*	2018	51.1
6	69	Nasopharyngeal carcinoma	Ma Jun	*Lancet*	2019	168.9
7	62	Immunization with Epstein-Barr virus (EBV) peptide-pulsed dendritic cells induces functional CD8 + T-cell immunity and may lead to tumor regression in patients with EBV-positive nasopharyngeal carcinoma	Lin CL	*Cancer Research*	2002	11.2
8	57	Adoptive T-cell transfer and chemotherapy in the first-line treatment of metastatic and/or locally recurrent nasopharyngeal carcinoma	Toh HanChong	*Molecular Therapy*	2014	12.4
9	53	Adoptive transfer of autologous Epstein-Barr virus-specific cytotoxic T cells for nasopharyngeal carcinoma	Ng MH	*International Journal of Cancer*	2001	6.4
10	53	Adoptive transfer of EBV-specific T cells results in sustained clinical responses in patients with locoregional nasopharyngeal carcinoma	Gottschalk Stephen	*Journal of Immunotherapy*	2010	3.9

IF = impact factor.

### 3.6. Analysis of keywords

**Figure 4.**
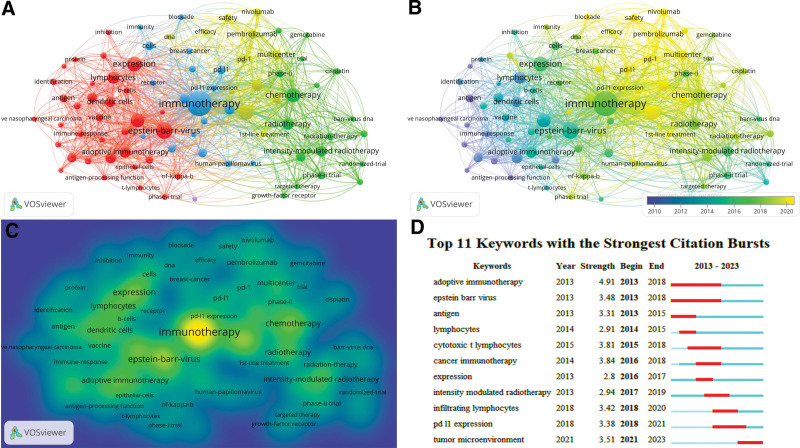
Analysis of keywords. Network map (A), overlay map (B), and density map (C) of keywords. Font display size is positively correlated with keyword frequency. In (B), purple represents early time, yellow represents near time. In (C), the brighter the color, the higher the average frequency of keywords. TOP 11 keywords with the strongest citation bursts (D).

It is important to analyze the keywords in the literature, which can reflect the focus of the article, and conducting many analyses can help us understand the hotspots of the related research and analyze the trend of its development. VOSviewer was used to visualize the keyword network, as shown in Figure 4. There are mainly red, blue, green, and yellow in Figure 4A, which roughly differentiates several research directions of NPC immunotherapy by color. Red is mainly related to basic research on NPC immunotherapy, and the main keywords include EBV, lymphocytes, expression, and adoptive immunotherapy; blue keywords mainly include immunotherapy, pd-l1 expression, prognosis, and survival. This group mainly focuses on the prognosis of immunotherapy for nasopharyngeal cancer; the green topic is other treatment modalities for NPC, and the main keywords include chemotherapy, radiotherapy, and concurrent chemotherapy. Smaller clusters, such as those represented in yellow, failed to form the major themes. In Figure 4B, purple represents an earlier occurrence, and yellow represents a more recent occurrence. In Figure 4C, the brighter yellow color represents a higher average number of times the keyword appeared, which to some extent reflects the research hotspots and direction shifts.

We used CiteSpace analysis to find that 11 frequent keywords appeared in 2013 to 2023, as indicated in Figure 4D. The blue line represents the time span between 2013 and 2023, and the red line represents the duration of each keyword burst. The dominant terms since 2018 include “infiltrating lymphocytes,” “pd-l1 expression,” and “tumor microenvironment.”

## 4. Discussion

### 4.1. General information

In the present study, we used WoSCC to search for bibliometric analyses of literature published between January 1, 2000 and September 1, 2023 related to immunotherapy for nasopharyngeal cancer. This study included 510 publications in 250 journals from 782 institutions in 51 countries/regions.

The results showed that the number of publications on NPC in this aspect of immunotherapy showed an upward trend, which shows that this research direction has attracted much attention in recent years. The various time periods require careful consideration. For the first time in 2017, the number of published articles increased from 11 in 2016 to 31 in 2017 with a yearly increase of 181.82%, which may be related to the fact that in 2017, the *Journal of Clinical Oncology* published for the first time on immunotherapy for NPC the KEYNOTE-028 study.^[18]^ The second is the 100% annual growth rate in the number of publications in 2019, which is considered to be related to the publication of the results of the phase Ib study of carrelizumab rate (camrelizumab) for NPC in *Lancet Oncology* in late 2018^[19]^ and the first publication of the preliminary results of the phase II registration study of teraplizumab, POLARIS-02, in ASCO in 2019 results are relevant. This is followed by an increase in the number of articles from 33 in 2020 to 50 in 2021 (51.52% annual growth rate), a phenomenon considered to be related to the approval of the anti-PD-1 monoclonal antibody teraplizumab in 2021 for treating patients with R/M NPC who did not respond to second line or higher systemic treatment.^[20]^ Thus, NPC has entered a new era of immunotherapy, with a continued increase in the quantity of literature in this field by 2022 (50.00% annual growth rate).

In Figure 2B, China and the United States account for 74.71% (381/510) of the total number of publications. China ranks first in the number of articles, which may be related to the fact that China has the highest incidence of NPC in the world, accounting for half of the global nasopharyngeal cancer cases.^[21]^ Although the United States was second in terms of the number of publications, the national collaborative overlay map (Fig. 3A) and centrality index analysis (Fig. 2B) showed that the United States was at the core of the network, suggesting that it plays the role of an intermediary in the network. This means that it has the greatest impact on the publishing industry, followed by Italy and China. The distribution of institutions and countries is similar, with 6 of the top 11 organizations located in China, followed by 2 organizations in the United States.

The top 9 journals published 107 publications, accounting for only 20.98% of the total literature. *Frontiers in Oncology* ranked first with regard to publications (n = 22), followed by *Frontiers in Immunology* (n = 19), which shows that these journals pay attention to research about immunotherapy for NPC, which can be useful for subsequent researchers in the selecting journals when submitting papers related to nasopharyngeal cancer immunotherapy.

Chen Qiuyan led the pack in terms of most published works, closely followed by Mai Haiqiang, Rooney Clionam, and Zeng Yixin. Comoli P stands out as the most prominent author among those who are cited together, followed closely by Khanna R and Bollard CM in terms of citation numbers. The fact worth mentioning is that Khanna R and Smith C are among the top 10 authors in terms of both publications and citations. This shows that they are accomplished by scholars in the field, and their team will be preferred by researchers for collaboration.

Among the top 10 highly cited articles, it mainly dealt with 2 treatment modalities: autologous transfusion of EBV-specific cytotoxic T cells (EBV-CTL) and injection of immune checkpoint inhibitors. “Treatment of nasopharyngeal carcinoma with Epstein-Barr virus-specific T lymphocytes” was the most cited document, and its main conclusion was that the autologous back infusion of EBV-CTL treatment modality in EBV-positive advanced NPC is feasible, appears to be safe and may have significant antitumor activity.^[17]^ Of these 10 publications, 6 were on EBV-CTL adoptive immunotherapy, with a publication spanning 2001 to 2014; 3 were clinical trials of nabulizumab, pembrolizumab, and camrelizumab in recurrent/metastatic NPC, published in 2017 and 2018, and 1 was an NPC review that was published in 2019. The cited articles show a shift in the immunotherapy approach for NPC, from targeting EBV to immune checkpoint inhibitors. By examining relevant academic papers, we can gain insights into the current state of research and the evolving patterns within this domain.

China produced 5 out of the top 10 co-cited articles, with the United States producing 2. China has led the world in research publications, with 6 of the top 10 institutions in the country. Furthermore, China has the highest contribution of highly co-cited literature, indicating its significant involvement in nasopharyngeal cancer research.

Based on the above analysis, we can know that: nasopharyngeal cancer treatment has entered the immune era, bringing new hope to patients; China and the United States have a pivotal role in nasopharyngeal cancer immunotherapy, and most of the authors and literature with significant impact in this field come from these 2 countries.

### 4.2. Current and future trends

An examination of keywords can provide insights into the evolving trends of studies within this area. As depicted by the findings displayed in Figure 4, studies related to immunotherapy for NPC have mainly focused on adoptive immunotherapy for EBV-CTLs, radiotherapy, and PD-L1/PD-1 therapy. The research trend is shifting from over-the-counter immunotherapy, intensity-modulated radiotherapy, and concurrent radiochemotherapy to antitumor activity, PD-L1/PD-1, and the tumor microenvironment. This is similar to the results of the 11 frequent keywords analyzed with CiteSpace (Fig. 4D), such as the frequent keywords “adoptive immunotherapy,” “epstein barr virus,” “cytotoxic t lymphocytes,” “intensity modulated radiotherapy,” “pd l1 expression,” and “tumor microenvironment.”

In 1995, the effectiveness of EBV-CTL adoptive immunotherapy in bone marrow transplant recipients was proven with positive outcomes.^[22]^ A strategy to activate in vivo CTL specific for EBV antigens expressed in NPC cells for the treatment of NPC was documented in 1998.^[23]^ In 2001, the utilization of adoptive immunotherapy for NPC patients was documented in a published paper about EBV-CTL, and the findings justified the need for additional research on EBV as a potential target for immune intervention in NPC.^[24]^ This was the first report on the use of EBV-CTL adoptive immunotherapy in NPC patients and became 1 of the top 10 highly co-cited documents on NPC immunotherapy. Data on the use of EBV-CTL adoptive immunotherapy for the treatment of a patient were reported in 2004, and the results showed that EBV-CTL was safe and may exert specific killing effects on NPC tumor cells in vitro and induce antitumor effects.^[25]^ Subsequently, in 2005, *Blood* published a report on the results of a clinical trial on the treatment of NPC with EBV-CTL, namely “Treatment of nasopharyngeal carcinoma with Epstein-Barr virus-specific T lymphocytes,” which enrolled 10 patients and confirmed the feasibility and safety of the therapy.^[17]^ This article thus became the most co-cited article in the literature related to NPC immunotherapy of NPC. Subsequently, EBV-CTL adoptive immunotherapy has become a hot research topic in NPC immunotherapy and several clinical trials on this therapy have been published. Notably, 1 study showed that the response rate of R/M NPC patients increased to 71.4% by combining chemotherapy and EBV-CTL adoptive immunotherapy.^[26]^ A multicenter phase II randomized controlled trial is ongoing to evaluate EBV-CTL immunotherapy after gemcitabine and carboplatin treatments (NCT02578641).

The frequent keywords “infiltrating lymphocytes” and “pd l1 expression” in 2018 had possible connections with the KEYNOTE-028 study (2017) as well as the NCl-9742 and CAPTAIN studies (2018), which brought significant advancements in the field. The efficacy and safety of pembrolizumab were investigated in 27 patients with NPC in a nonrandomized, multicohort, Phase Ib KEYNOTE-028 study.^[18]^ The results of the study showed an objective remission rate of 25.9%, a 6.5-month median progression-free survival, and a median OS of 16.5 months were observed during a median follow-up period of 20 months. The results of treatment were related to the expression of PD-L1. The effects of programmed death-1/programmed death ligand-1 inhibitors are mainly based on the active immunity of autoimmune cells, especially tumor-infiltrating lymphocytes; therefore, research has been focused on the tumor microenvironment, as shown in Figure 4D, which has received much attention in nasopharyngeal cancer immunotherapy since 2021. Many immune cells can be found in this tumor, such as T cells and B cells,^[9]^ this shows that NPC has a good immune microenvironment. An in-depth study of the tumor microenvironment may help predict the efficacy of treatments and interventions and may lead to the discovery of new research methods that are worth looking forward to.

The application of ICI in NPC has provided a new direction for the treatment of NPC. Since the report of the KEYNOTE-028 study in 2017, ICI have become a research hotspot in immunotherapy for NPC. As shown in Figure 4D, the keyword “pd-l1expression” began to appear frequently in 2018. The 2-year follow-up results of the CHECKMATE141 study^[27]^ showed that the median OS time of the nivolumab group and the standard treatment group was 7.7 months and 5 minutes, respectively, and the 2-year survival rate was nearly 3 times that of the standard treatment group (16.9% vs 6%). Based on this result, in 2019, China’s National Medical Products Administration (NMPA) formally approved nivolumab as the first immunosuppressant in China to treat patients with PD-L1-positive head and neck squamous cell carcinoma during or after platinum-containing regimens. In the same year, based on the 4-year follow-up results of Keynote 048,^[28]^ the FDA approved pembrolizumab combined chemotherapy as the first-line treatment for PD-L1-positive R/M head and neck tumors. Toripalimab showed stable therapeutic efficacy in second line and above regimens in clinical trials.^[29,30]^ Toripalimab was approved by NMPA in February 2021 and became the first approved anti-PD-1 monoclonal antibody drug in the world for the treatment of previously failed second line or above systematic treatment of R/M NPC. The results of several clinical trials^[19,31]^ showed that camrelizumab combined with gemcitabine and cisplatin was safe and effective. In April 2021, NMPA approved camrelizumab in combination with gemcitabine and cisplatin for the first-line treatment of R/M NPC, the world’s first approved first-line immunotherapy regimen for R/M NPC.

In this study, we can learn that the focus of immunotherapy for nasopharyngeal cancer has shifted from passive immunotherapy, such as adoptive cell transfer therapy, to active immunotherapy, such as immune checkpoint inhibitors. We are pleased that immunotherapy for nasopharyngeal cancer has achieved considerable success, but it is undeniable that many difficulties and challenges remain. Well-known biomarkers, such as PD-L1 expression and tumor mutation burden, have not yet been shown to have clear clinical predictive value for immunotherapy in NPC. More studies are needed to find suitable markers to predict the response and efficacy of immunotherapy in order to maximize the personalized therapeutic approach for NPC. Therefore, further studies of the NPC tumor microenvironment to understand the molecular and cellular drivers of immune escape to overcome immunotherapeutic resistance are important and may identify new therapeutic directions to improve the outcome of NPC patients.

Bibliometrics is a quantitative research method used to analyze scholarly publications and their impact within a specific field of study. It possesses the potential to illustrate the progression of scientific understanding and its underlying relationships while simultaneously bringing to light numerous intricate connections concealed within groups of information.^[32,33]^ Tumor immunotherapy has recently become a relatively new and promising therapeutic method. This can result in promising clinical outcomes, but tumor heterogeneity and diversity are inevitable. Scholars can comprehend specific patterns of knowledge by understanding these intricate chains of knowledge. According to our bibliometric analysis, immunotherapy for nasopharyngeal cancer is a current research priority, with studies focusing on immune checkpoint inhibitors and the tumor microenvironment.

## 5. Strengths and limitations

To the best of our knowledge, this represents the first comprehensive examination of literature and patterns in NPC immunotherapy, providing detailed information for physicians and scholars in the field. Simultaneously, we employed diverse bibliometric software tools to analyze research trends from various perspectives. This study has some limitations. First, the studies we used may not have been exhaustive. First, the data we analyzed only came from WoSCC, while excluding data from other prominent search engines, such as PubMed, Ovid, and Embase. Second, we limited the language of the publications to English, which led to language bias. Thus, this study may not fully represent the current state of immunotherapies for NPC. Third, given the significantly low rate of citations, recent exceptional publications may fail to garner the recognition that they truly merit. This finding highlights the significance of incorporating new findings into future research. Finally, although this study only examined papers published in the field of NPC immunotherapy from January 1, 2000 to September 1, 2023, excluding documented publications from previous years, and the data for 2023 are incomplete, new findings in the future could influence the outcomes.

## 6. Conclusion

In this study, we used bibliometric methods to summarize and analyze trends in NPC immunotherapy research. Our survey revealed a rising pattern in the overall quantity of literature, suggesting a growing fascination among researchers in this area. China has contributed the most to the NPC research. Recently, there has been a growing research interest in the field of NPC regarding programmed death-1/programmed death ligand-1, infiltrating lymphocytes, and the tumor microenvironment, suggesting that immunotherapeutic research in NPC has focused on immune checkpoint inhibitors and the tumor microenvironment.

## Author contributions

**Conceptualization:** Huanhuan Xie, Wenjing Liu, Mi Yang.

**Data curation:** Huanhuan Xie, Wenjing Liu.

**Formal analysis:** Huanhuan Xie.

**Funding acquisition:** Huanhuan Xie, Mi Yang.

**Investigation:** Huanhuan Xie.

**Methodology:** Huanhuan Xie.

**Project administration:** Mi Yang.

**Software:** Huanhuan Xie.

**Supervision:** Mi Yang.

**Validation:** Wenjing Liu.

**Visualization:** Huanhuan Xie, Wenjing Liu.

**Writing – original draft:** Huanhuan Xie.

**Writing – review & editing:** Huanhuan Xie, Wenjing Liu, Mi Yang.
